# Absent pulmonary valve syndrome with tetralogy of Fallot and associated dextrocardia detected at an early gestational age of 26 weeks

**DOI:** 10.4103/0971-3026.43841

**Published:** 2008-11

**Authors:** Alpa H Bharati, Ajita Naware, Suleman A Merchant

**Affiliations:** Department of Radiology, Lokmanya Tilak Municipal General Hospital, Sion, Mumbai, India

**Keywords:** Pulmonary valve, antenatal US, dextrocardia, tetralogy of Fallot

## Abstract

Absent pulmonary valve syndrome is a rare congenital anomaly, usually seen in association with a ventricular septal defect. It has been reported to occur in 3–6% of cases of tetralogy of Fallot. Absence of the pulmonary valve results in a dilated main pulmonary artery, which can be seen as a cystic, pulsatile, paracardiac lesion on antenatal USG. Such a lesion, though rare, can easily be detected. We report a case of this rare anomaly which was present in association with a ventricular septal defect, tetralogy of Fallot, and dextrocardia. The case was detected at 26 weeks of gestation.

Tetralogy of Fallot (TOF) occurring along with the absent pulmonary valve syndrome (APVS) is a rare congenital cardiac malformation. APVS is detected in 3-6% of TOF patients. The prognosis depends on the respiratory complications.[1] When APVS is associated with a ventricular septal defect, the physiologic and anatomic repercussions affect both ventricles, and cardiac performance can be critically impaired.[2]

We report a case of APVS that was detected by USG at 26 weeks of gestation; there was an associated ventricular septal defect along with an absent ductus arteriosus and dextrocardia, a feature not seen with this syndrome till date as per our literature search. The aneurysmally dilated pulmonary artery had resulted in severe right bronchial compression, right lung hypoplasia with fetal cardiac failure, and fetal hydrops, all of which culminated in early fetal demise.

## Case Report

**Figure 1:**
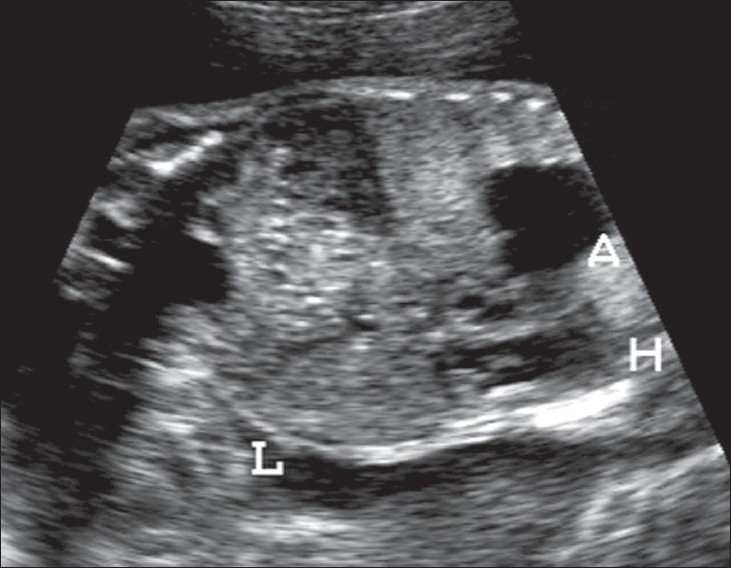
Coronal section of the fetal thorax and abdomen shows the heart (H) on the right side with hypoplasia of the right lung. Also depicted are the liver (L) and aneurysmal dilatation of the pulmonary artery (A), which is seen as a cystic lesion

**Figure 2:**
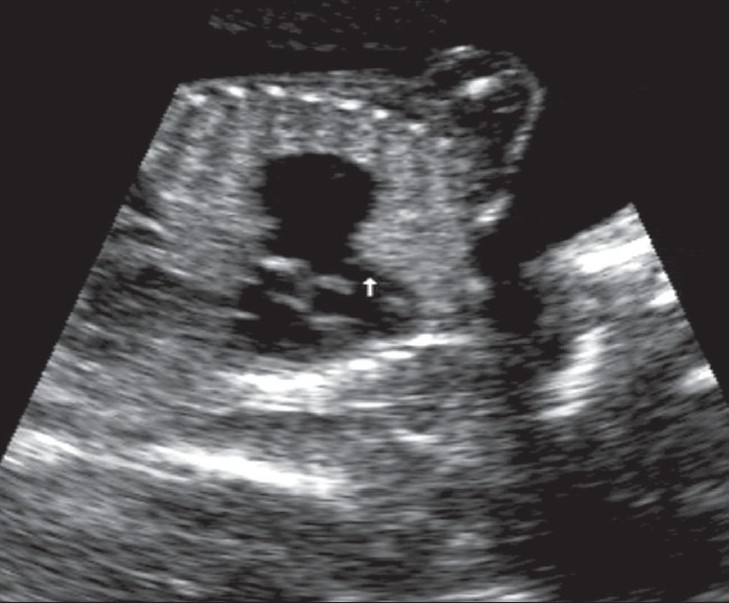
An oblique coronal image of the fetal thorax reveals the aneurysmally dilatated main pulmonary artery and absence of pulmonary valve echoes (arrow)

**Figure 3:**
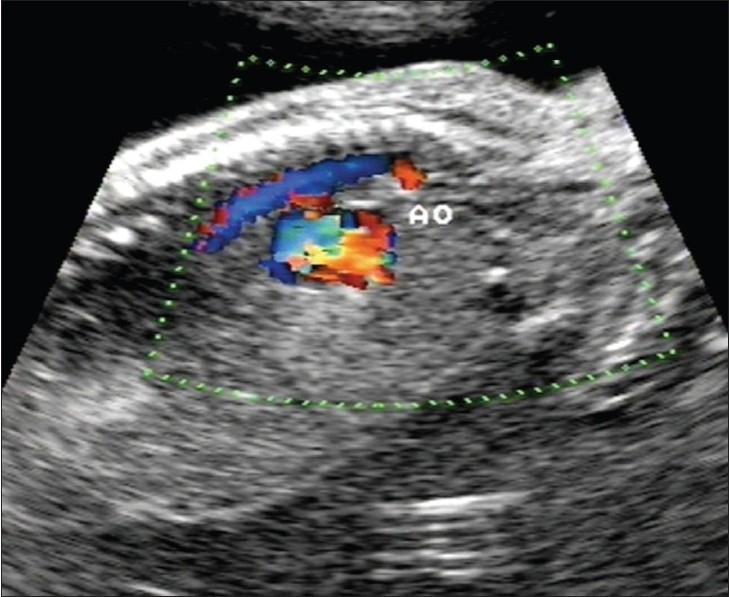
Color Doppler imaging in the left parasagittal plane of the fetal thorax shows the aorta (ao) and the dilated pulmonary artery, but no ductus arteriosus

**Figure 4:**
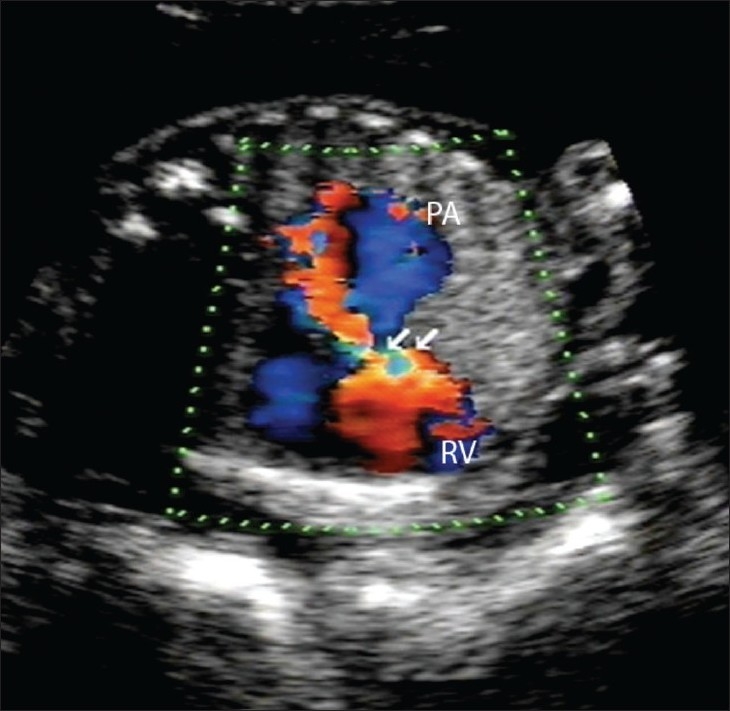
An axial color Doppler image of the right ventricular outflow tract (arrows) shows the dilated pulmonary artery (PA) and the right ventricle (RV) with a mixed hue of colors suggesting to-and-fro random flow between the right ventricle and the pulmonary artery

**Figure 5:**
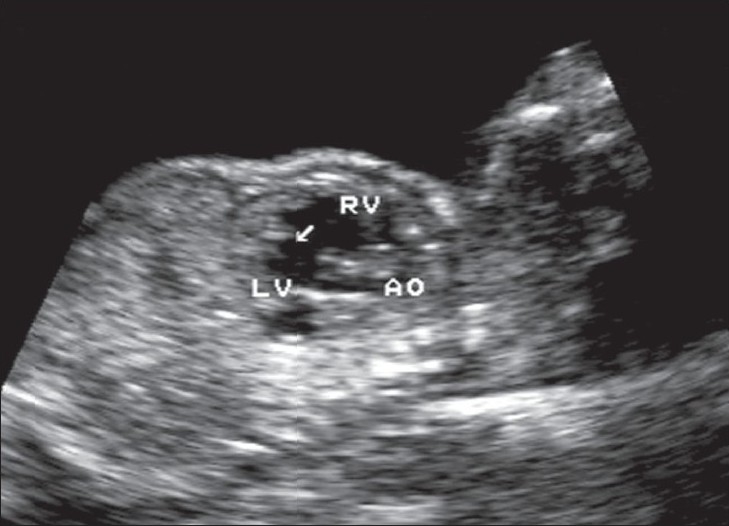
Sagittal image of the fetal thorax reveals a large subaortic VSD (arrow) between the right ventricle (RV) and the left ventricle (LV). Aorta (AO) is seen arising from the LV

**Figure 6:**
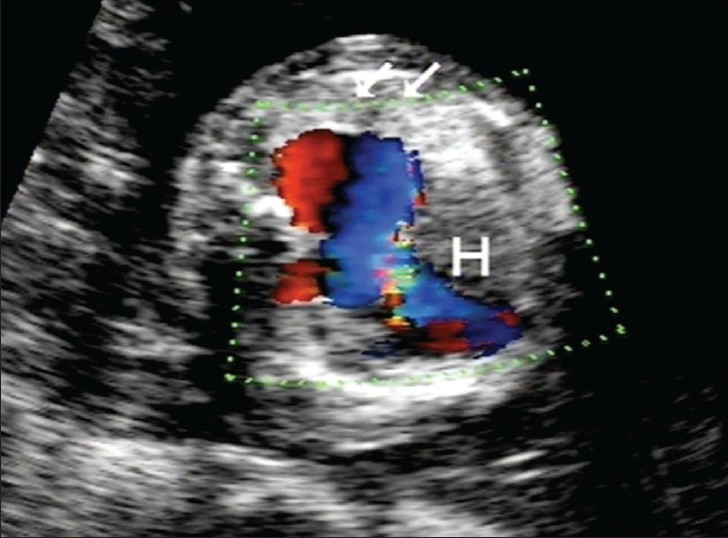
Axial image of the fetal thorax reveals the enlarged right ventricle and dilated main pulmonary artery. Also noted is the relatively preserved left lung (arrows) with no obvious pulmonary tissue on the right, the space being occupied by the heart (H)

A 20-year-old primigravida presented for routine prenatal USG scanning. There was a single fetus of about 26 weeks' gestation and severe maternal polyhydramnios. The fetus showed a pulsatile cystic lesion located near the heart. We therefore performed detailed fetal echocardiography, which revealed abdominal situs solitus (liver on the right side and fundic bubble on the left side); there was also dextrocardia with atrial situs solitus [Figure 1]. The superior and inferior vena cava drained normally into the right atrium. Atrioventricular concordance was noted. The tricuspid and mitral valves were normal. The right ventricle (RV) was seen to open into the pulsatile cystic lesion, which was thus confirmed to be the dilated main pulmonary artery along with the right and left pulmonary arteries. The pulmonary annulus showed no pulmonary valve (p-valve) echoes [Figure 2]. The ductus arteriosus was not visualized [Figure 3]. There was back-and-forth flow across the RV and the dilated pulmonary artery during systole and diastole of the RV [Figure 4]. The aorta was seen arising from the left ventricle (LV). A large subaortic ventricular septal defect (VSD) was seen [Figure 5]. The fetus also showed mild ascites and hypoplasia of the right lung [Figure 6]. Other systems were unremarkable. Thus, a diagnosis of TOF with dextrocardia and absent p-valves, along with absent ductus arteriosus and hypoplasia of the right lung, was made. The fetus was stillborn at 29 weeks of gestation and autopsy findings confirmed the USG and fetal echocardiographic findings of TOF with APVS.

## Discussion

APVS is a complex syndrome comprising dysplasia / absence of pulmonary valvular leaflets, with resultant regurgitation and dilatation of the main and branch pulmonary arteries.[3] The majority of these cases present with a VSD and features of TOF. The aneurysmal dilatation of the pulmonary artery often results in compression of the bronchial tree and esophagus, with consequent bronchomalacia and polyhydramnios.

Volpe *et al.* studied 21 fetuses with APVS for their associations and outcomes. Their study reveals an association of this syndrome with microdeletion of chromosome 22q11 in 25% of cases. They also suggest that bronchomalacia is commonly associated with cardiomegaly and dilatation of the pulmonary artery and results in poor prognosis.[4]

APVS, in the absence of VSD, is uncommon. As reported by Yeager *et al.*, most of the cases presenting with an intact ventricular septum commonly reveal a patent ductus arteriosus, with relatively small pulmonary arteries and associated tricuspid atresia.[2] According to their study, the free communication between the ventricles and the aorta causes the blood flow to the atria to be reduced, while there is increase in the ventricular end diastolic pressure; this may in turn affect the cardiac function and the development of the atrioventricular valve. Yeager *et al.* also suggest that in the presence of a VSD these changes affect both the ventricles, thereby resulting in a poor prognosis,[2] as seen in our case. 

A grossly dilated pulmonary artery can cause compression of the tracheobronchial tree and the esophagus. This obstructs the normal amniotic fluid circulation, causing polyhydramnios. As stated by Callan *et al.*, the presence of polyhydramnios may indicate a poor prognosis.[5]

In our study, the fetus had a grave prognosis as there was absence of the ductus arteriosus and a large VSD, which had resulted in pressure changes in both the ventricles with severe pulmonary arterial dilatation and early cardiac failure. The diagnosis was obtained on antenatal USG and echocardiography at the relatively early stage of 26 weeks of gestation. These findings were consistent with the findings reported by others with regard to APVS with absent ductus arteriosus and VSD. 

## Conclusion

We conclude that when a paracardiac cystic, pulsatile lesion is seen in the fetus in utero, APVS is an important differential diagnosis and other features associated with the syndrome, such as TOF and the presence or absence of the ductus arteriosus should be looked for. We report a rare association of dextrocardia in our case of APVS with TOF.
